# Efficacy of Transversus Abdominis Plane Block in Patients After Laparoscopic Radical Cervical Cancer Surgery

**DOI:** 10.3389/fsurg.2022.674987

**Published:** 2022-04-01

**Authors:** Xiaoyu Ma, Yi Gao, Jing Wang, Zhen Wu, Huasu Shen, Ping Wang

**Affiliations:** ^1^Department of Anesthesiology, The Fourth Hospital of Shijiazhuang, Shijiazhuang, China; ^2^Department of Anesthesiology, Central Hospital of Jiaozuo Coal Industry Group, Jiaozuo, China; ^3^Department of Anesthesiology, The Fourth Hospital of Hebei Medical University, Shijiazhuang, China

**Keywords:** transverse abdominal muscle plane block, laparoscopy, radical cervical cancer, pain, immunity

## Abstract

**Objective:**

This study aimed to evaluate the efficacy of transversus abdominis plane block (TAPB) in patients after laparoscopic radical cervical cancer surgery.

**Methods:**

A total of 120 patients with cervical cancer who underwent laparoscopic radical resection in the hospital from January 2019 to January 2020 were selected and concurrently assigned to either patient-controlled intravenous analgesia (PCIA) (Control group) or PCIA plus TAPB (Observation group) according to different methods. The visual analogscale (VAS), Bruggemann comfort scale (BCS), immune function indicators, hemodynamics, analgesia indicators, and postoperative recovery time were recorded and compared.

**Results:**

The Observation group had a lower VAS score and a higher BCS postoperatively compared with the Control group, and the difference was statistically significant. After the operation, immune function indexes of patients in the two groups were declined, and the difference was statistically significant. At 76 h after operation, the immune function indexes of the Control group were lower than the Observation group, and the difference was statistically significant. After the operation, the Control group obtained a higher mean arterial pressure (MAP) at extubation, and the difference was statistically significant. The Observation group outperformed the Control group in terms of analgesia indicators and postoperative recovery time, and the difference was statistically significant.

**Conclusion:**

TAPB can enhance the analgesic effect of patients after laparoscopic radical resection of cervical cancer, stabilize their physical signs, has little effect on the patient's immune function, with a high safety profile.

## Introduction

Laparoscopy has gained increasingly extensive application in recent years, which is largely attributed to its small incision and rapid recovery. However, distinct pain elicited by postoperative abdominal distension, incision, and injuries to the uterus and other parts of the body still exist (1). Without timely and effective analgesic management, it may progress from an acute type to a chronic one (2). The need for pain relief by analgesics is associated with 73 to 80% of patients after laparoscopic surgery, and opioids are required among the rest 20%. Laparoscopic surgery is the conventional therapy for patients with cervical cancer, featured by simple operation and less surgical trauma, but has a high requirement of anesthesia and analgesia (1–3), which necessitates efficient anesthesia management to alleviate the postoperative pain and promote recovery. With the continuous optimization of ultrasound technology, transversus abdominis plane block (TAPB), as a routine anesthesia technique (4–7), has upgraded as visual anesthesia and captured great attention in clinical anesthesia research. Nonetheless, TAPB in minimizing postoperative pain and protecting immune function is marginally studied, and the mechanism of TAPB affecting immune function remains elusive. Therefore, this study aimed to investigate the actual effect of TAPB in analgesia in patients after laparoscopic radical resection of cervical cancer, and its impact on immune function. The results are shown as follows. The innovation of this study lies in the use of a new analgesic model to analyze the efficacy of patients to provide data for future clinical prognosis improvement.

## Materials and Methods

### General Material

A power analysis was conducted according to the study by Hui et al. (8), which reported that the average visual analog scale (VAS) score after laparoscopic radical resection of cervical cancer was 4.04 (*SD* ± 0.73), the average VAS score after ultrasound-guided transversus abdominis plane block was 1.78 (*SD* ± 0.64), so we believed that the 40% difference might be of clinical significance. In order to obtain 60% power, α = 0.05, β = 0.1, it was calculated that the sample size of each group was 50 cases. Considering the dropout rate of 20%, a total of 120 patients undergoing laparoscopic radical resection of cervical cancer were included in our study from January 2019 to January 2020, with 60 cases in each group. The baseline data were well balanced between the two groups (*P* > 0.05), Table 1. The study was approved by the hospital ethics committee and conducted under supervision, and all patients signed informed consent.

**Table 1:** General data of patients.

	**Average Age** **($\bar{x}$ ± s, year)**	**Height** **($\bar{x}$ ± s, cm)**	**Weight** **($\bar{x}$ ± s, kg)**	**ASA Grading (** ***n, %*** **)**	
				** < II**	**≥III**
Observation group (*n* = 60)	47.52 ± 7.45	160.56 ± 8.65	55.21 ± 6.12	32 (53.33)	28 (46.67)
Control group (*n* = 60)	47.56 ± 7.89	160.54 ± 7.89	55.23 ± 6.71	33 (55.00)	27 (45.00)
*χ^2^/t*	0.029	0.013	0.017	0.034	
*P*	0.977	0.990	0.986	0.855	

### Inclusion Criteria

(a) Cervical cancer diagnosed by preoperative hysteroscopy and postoperative pathological examination; (b) Laparoscopic radical resections were scheduled, and there were no contraindications to surgery; (c) Patients with no heart and lung diseases; (d) Patients with no history of abdominal surgery.

### Exclusion Criteria

(a) Patients with allergies to the drug applied in the trial; (b) Patients with any psychiatric or cognitive disorder impairing cognition or the inability to communicate; (c) Patients with other major organic diseases; (d) Patients who received analgesia therapy within 7 days prior to the study; (e) Allergies to anesthetics.

### Methods

All patients underwent conventional treatment. The Control group received patient-controlled intravenous analgesia (PCIA), and the Observation group received PCIA plus TAPB. Details are described below.

#### Anesthesia

After entering the operating room, the right internal jugular vein and left radial artery are punctured for catheterization. The arterial blood pressure, finger pulse oxygen saturation, and other physical data of the patient were monitored. Sedation with intravenous 0.05 mg/kg midazolam (Jiangsu Nhwa Pharmaceutical Co., Ltd., Xuzhou, Jiangsu, National Drug Approval H20143222), 0.4 μg/kg sufentanil (Yichang Renfu Pharmaceutical Co., Ltd., Yichang, Hubei, National Drug Approval H20054172), and 0.9 mg/kg disoprofol (Fresenius Kabi AB, Bad Homburg, Germany, National Drug Approval J20080023) were premedicated, followed by mechanical ventilation and end-expiratory carbon dioxide recording. Anesthesia was maintained by a continuous infusion of 3.5 mg/kg/h propofol and 10 μg/kg/h remifentanil (Yichang Renfu Pharmaceutical Co., Ltd., Yichang, Hubei, National Drug Approval H20030197). Muscular flaccidity was maintained by intermittent intravenous injection of Cisatracurium Besilate (Jiangxi Shimei pharmaceutical Co., Ltd., Fuzhou, Jiangxi, National Drug Approval H20083362).

#### Control Group

Carbon dioxide pneumoperitoneum was achieved with intra-abdominal pressure maintained lower than 15 cm H_2_O. All patients received 150 ml i.v. of PCIA solution immediately after extubation. PCIA solution contained sufentanil 140 μg/kg in normal saline 148 ml. The PCA pump was programmed with no background continuous infusion to deliver a single dose of 2 ml, no more than 24 ml, with the time set to 15 min.

#### Observation Group

After mechanical ventilation and skin disinfection, the probe was placed between the costal margin and the iliac crest to clearly display the internal and external oblique muscles, transverse abdominis muscles, and TAP. This was done using the ultrasonic probe (German Nicolet, TC8080) set to 45 MZ and the frequency of 8 MHz, followed by the injection of 18 ml 0.35% ropivacaine between the transverse abdominis muscles and internal oblique.

### Outcome Measures

#### Primary Endpoints

(a) Visual analog scale (VAS) is a 10-point sliding scale, on which “0” means “no pain” and “10” means “unbearable severe pain” (9). The VAS score was recorded at 3, 12, 24, 48, and 72 h postoperatively, respectively.
(b) Analgesia index. The amount of sufentanil and times of analgesia pump pressure were recorded within 1 day after surgery (10).

#### Secondary Endpoints

(a) Postoperative recovery time refers to the time to gastrointestinal recovery and getting out of bed.
(b) Bruggemann comfort scale (BCS) was used to evaluate the presence of pain in patients during rest, coughing, or deep breathing (11). The closer to 0, the more obvious the pain. The BCS was recorded at 3, 12, 24, 48, and 72 h postoperatively, respectively.
(c) Immune Function. Fasting venous blood was collected from all patients for the determination of immune function indexes using flow cytometry. At 10 min before operation (T0), after the operation (T1), and 76 h after operation (T2), CD3^+^, CD4 ^+^, and CD4^+^/CD8^+^ were determined and analyzed.
(d)Hemodynamics. The mean arterial pressure (MAP) was measured at extubation in the two groups.

### Statistics Process

All data analyses were carried out using SPSS version 20, and Graphics rendering was carried out using GraphPad Prism version 8. Measurement data conforming to a normal distribution were expressed as (x ± s), independent samples *t*-test was used for comparison between two groups, paired-samples *t*-test was used for comparison within groups, one-way analysis of variance was used for comparison of three groups, and Snk-q test was used for pairwise comparison. Counting data were expressed by frequency or composition ratio, and verified *via* χ^2^ test. Differences were considered statistically significant at *P* < 0.05.

## Results

### Comparison of VAS Scores

**Figure 1:**
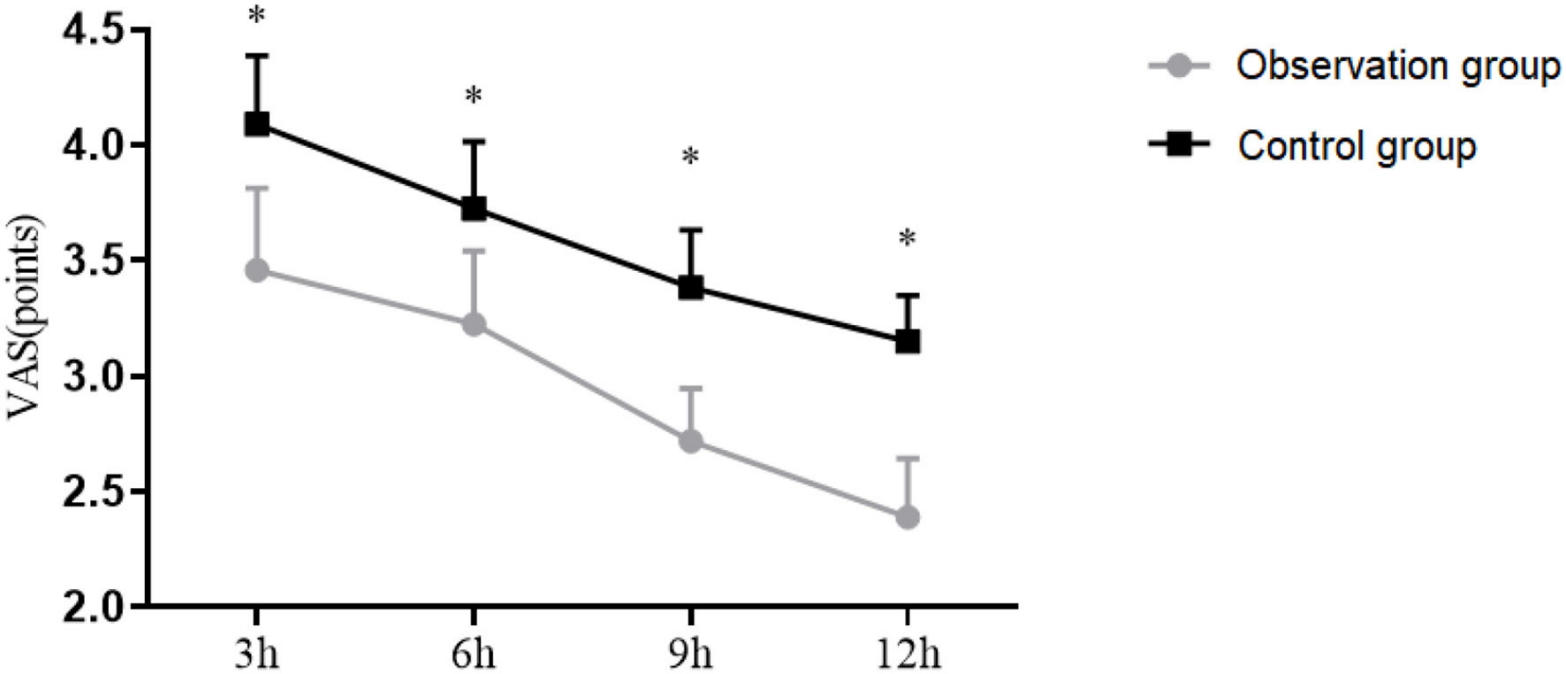
Visual analog scale (VAS) scores of patients. The transverse axis was time after surgery (3, 6, 9, and 12 h), and the vertical axis was VAS score. *P < 0.05.

The VAS scores at 3, 6, 9, and 12 h postoperatively in Observation group was (3.21 ± 0.5), (3 ± 0.45), (2.56 ± 0.32), (2.21 ± 0.36). The VAS scores at 3, 6, 9, and 12 h postoperatively in Control group was (3.88 ± 0.42), (3.52 ± 0.41), (3.21 ± 0.35), (3.01 ± 0.28). The VAS scores at 3, 6, 9, and 12 h postoperatively in the Observation group were significantly lower than those in the Control group (*P* <0.05), as shown in Figure 1.

### Comparison of Analgesia Related Indicators

The amount of sufentanil and the times of analgesic pump presses within 1 day postoperatively were lower in patients of the Observation group than those of the Control group (*P* < 0.05) as presented in Table 2.

**Table 2:** Analgesia-related indicators of patients ($\bar{x}$ ± s).

	**Dose of sufentanil (ug)**	**Pressure times of PCIA**
Observation group (*n* = 60)	35.11 ± 2.56	2.10 ± 0.45
Control group (*n* = 60)	40.02 ± 3.24	3.51 ± 0.86
*t*	9.210	11.252
*P*	<0.05	<0.05

### Comparison of Postoperative Recovery Time

Patients in the Observation group obtained a shorter time to gastrointestinal recovery and getting out of bed vs. the Control group (*P* <0.001), as presented in Table 3.

**Table 3:** Post-operative recovery time of patients ($\bar{x}$ ± s).

	**Gastrointestinal recovery**	**Get out of bed**
Observation group (*n* = 60)	20.21 ± 2.50	15.56 ± 3.20
Control group (*n* = 60)	26.21 ± 2.56	20.12 ± 3.12
*t*	12.989	7.903
*P*	<0.05	<0.05

### Comparison of BCS

**Figure 2:**
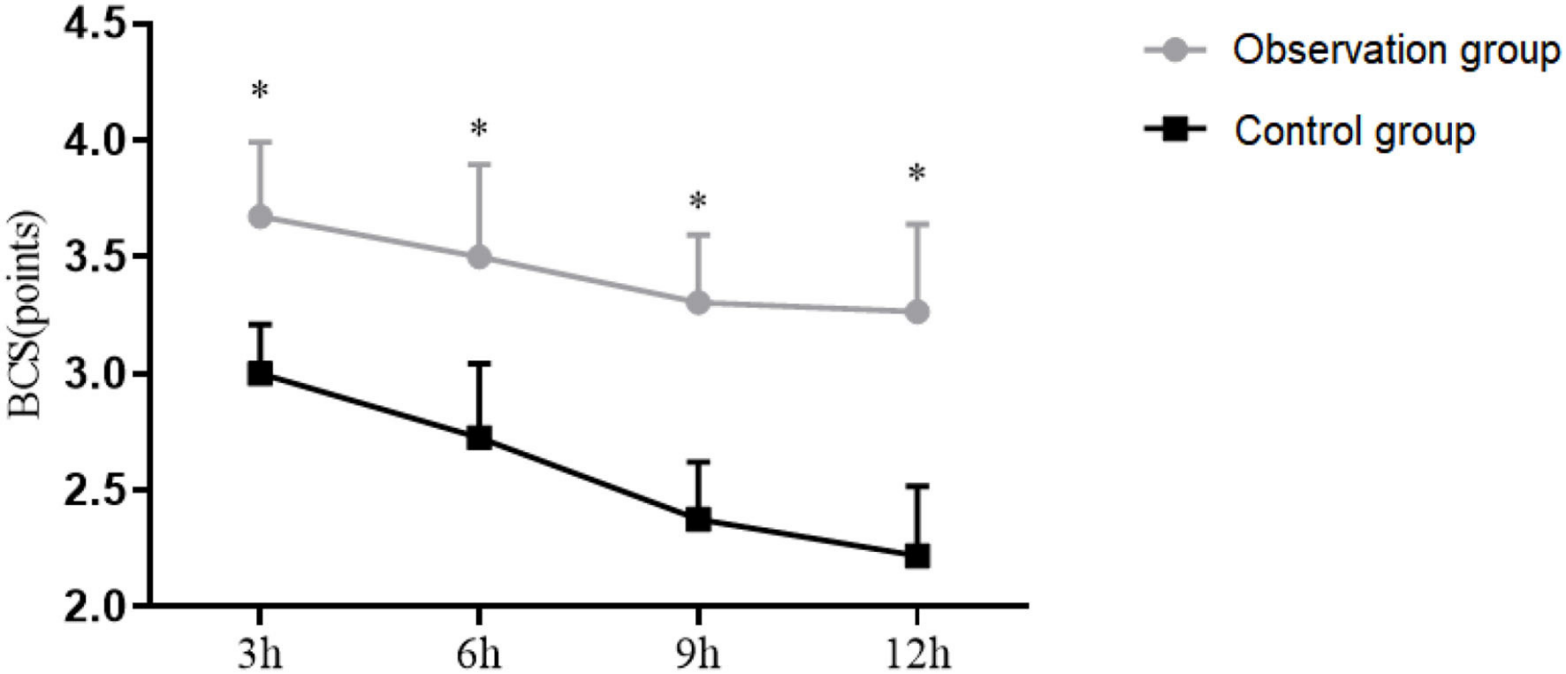
Bruggemann comfort scale (BCS) of patients. The transverse axis was time after surgery (3, 6, 9, and 12 h), and the vertical axis was BCS. ^*^P < 0.05.

The BCS at 3, 6, 9, and 12 h in Observation group was (3.45 ± 0.45), (3.22 ± 0.56), (3.1 ± 0.41), (3 ± 0.53). The BCS at 3, 6, 9, and 12 h in Control group was (2.85 ± 0.3), (2.5 ± 0.45), (2.2 ± 0.35), (2.01 ± 0.42). The BCS at in Observation group was and Control group was. The BCS at 3, 6, 9, and 12 h postoperatively in Observation group were significantly lower than those in Control group (*P* < 0.05), as shown in Figure 2.

### Comparison of Immune Function Indicators

The differences in CD3+, CD4+, and CD4+/CD8+ levels of the two groups at different time points were significant (*P* < 0.05). The Observation group had better CD3+, CD4+, and CD4+/CD8+ levels than the Control group (*P* < 0.05). See Table 4.

**Table 4:** Immune function indicators of patients ($\bar{x}$ ± s).

**Index**	**Time**	**Observation group**	**Control group**
CD3^+^(%)	T_0_	70.50 ± 7.51	70.51 ± 7.45
	T_1_	62.20 ± 7.89^a^^c^	56.56 ± 6.45^a^
	T_2_	68.21 ± 8.21^b^^c^	62.11 ± 7.54^a^^b^
*F*		17.778	57.672
*P*		0.000	0.000
CD4^+^(%)	T_0_	39.21 ± 4.56	39.28 ± 4.55
	T_1_	33.31 ± 6.89^a^^c^	31.12 ± 3.21^a^
	T_2_	37.89 ± 6.78^b^^c^	35.37 ± 3.56^a^^b^
*F*		15.108	68.637
*P*		0.000	0.000
CD4^+^/CD8+	T_0_	1.38 ± 0.23	1.37 ± 0.21
	T_1_	1.28 ± 0.18^a^^c^	1.20 ± 0.18^a^
	T_2_	1.35 ± 0.15^b^^c^	1.28 ± 0.10^a^^b^
*F*		4.401	15.069
*P*		0.014	0.000

*Compared with T0 time in the same group*,

a *P < 0.05; compared with T1 time in the same group*,

b *P < 0.05; compared with the control group at the same time*,

c *P < 0.05*.

### Comparison of Hemodynamics at Extubation

**Figure 3:**
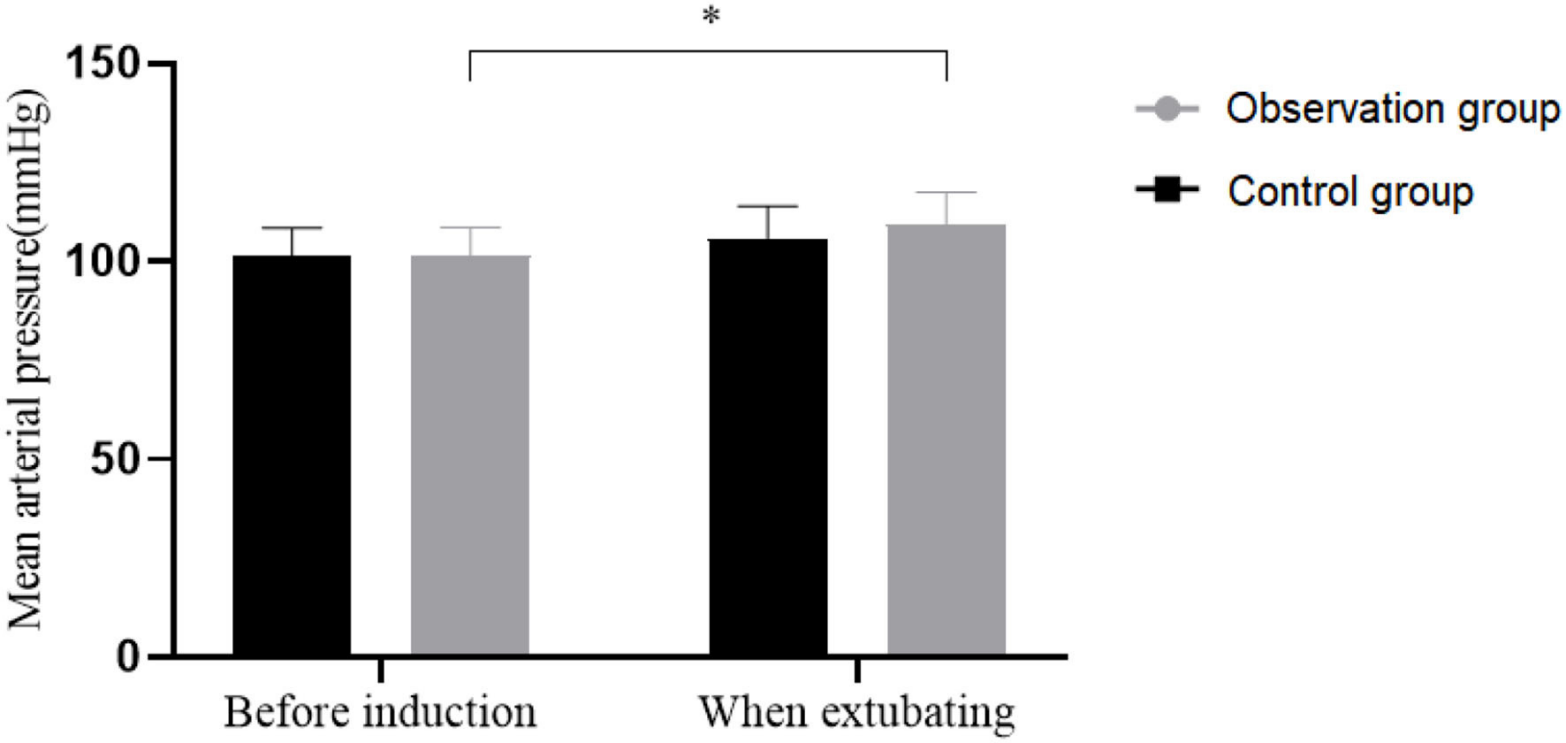
Hemodynamics of patients. The transverse axis was pre-induction (left) and extubation (right), and the vertical axis was MAP. *P < 0.05.

The MAP at pre-induction was (96.21 ± 10.11) mmHg in the Observation group and (96.2 ± 10.23) mmHg in the Control group. The MAP at extubation was (100.1 ± 11.45) mmHg in the Observation group and (103.56 ± 11.52) mmHg in the Control group. The Control group obtained a higher mean arterial pressure (MAP) at extubation (*P* < 0.05), and no significant difference was observed in the Observation group (*P* > 0.05). See Figure 3.

## Discussion

Multimodal analgesia is based on local analgesia, and nerve block techniques with ultrasound guidance have also gained momentum in recent years. The function of the anterior branch of the nerve from the thoracic spine (T7) to the lumbar spine (L1) is mainly to innervate the sensory nerves of the abdomen, muscles, and the wall peritoneum. These nerves pass through the lateral abdominal wall and the fascia contained in the internal oblique and transverse abdominal muscles to the anterior abdominal wall, where the nerves innervate the various muscle layers in the lateral abdominal wall with their dermal branches distributed in the skin of the region (4). The TAPB technique is a local blocking technique developed on this anatomical basis, in which anesthetics are administered in-plane to provide effective analgesia for patients undergoing open surgery.

Due to the dispersed distribution of nerves in the abdominal region through the lateral abdominal wall, TAPB requires a high dose of a local anesthetic to achieve blockade of multiple abdominal nerves, in most cases at a dose of 20–30 ml per side. Early TAPB was mainly localized by anatomy, and the success of the block was primarily defined by the presence of a sensation of penetration of the puncture needle through the internal and external oblique abdominal muscles, which, however, fails to ensure an efficiency predictably and has a poor safety profile. The TAPB procedure is currently performed under ultrasound guidance, using an in-plane technique that allows dynamic observation of the direction of needle insertion and needle tip position throughout the procedure, as well as clear monitoring of accurate drug entry and diffusion, thereby significantly improving the block completion rate.

Ultrasound-guided TAPB reduces the visual field blindness, clearly presents the nerves and surrounding tissues, which minimizes the incidence of complications caused by improper operation, to further attenuate the impact on the body and optimize the overall anesthetic effect (12–17). For patients undergoing laparoscopic radical resection of cervical cancer, the pain mainly stems from the abdominal incision and mostly intensifies after analgesic recovery. By blocking the anterior abdominal wall nerve, TAPB reduces the pain in the abdominal wall area, thereby achieving a promising and targeted analgesic effect (18–21).

In this study, postoperative VAS score and pressure times of PCIA in the Observation group were observed to be significantly lower than those in the Control group, and BCS was significantly higher than that in the Control group, indicating significant enrichment in a more ideal analgesic effect and higher postoperative comfort by TAPB plus PCIA. The postoperative recovery time in the Observation group was also shorter than that in the Control group, which was consistent with the research results of scholar Anna Buffey, the results also suggest that showed a promising analgesic effect and a rapid recovery of TAPB on patients undergoing radical cervical cancer surgery (22–24).

Surgical trauma and anesthesia may weaken the immune function of the patient after surgery. Herein, the immune function indexes of the Observation group at 76 h after surgery showed no significant difference compared with this before surgery but were significantly higher than that of the Control group, indicating a less affected immune function in patients receiving TAPB. In addition, no significant differences were found in mean arterial pressure in the Observation group between pre-induction and extubation, which confirmed a high safety profile of TAPB with fewer documented adverse reactions. The limitation of this study is that no follow-up study was conducted to assess the effect of TAPB on patients' prognosis and psychological status, and the trial will be further extended in the future to assess patients' quality of life and psychological status.

In conclusion, TAPB can enhance the analgesic effect of patients after laparoscopic radical resection of cervical cancer, stabilize their physical signs, has little effect on the immune function of patients, with a high safety profile. It might serve as a postoperative analgesia scheme for such patients. Nevertheless, some limitations merit attention in this study. The lack of analysis on the mechanism of transversus abdominis plane block anesthesia for postoperative analgesia after laparoscopic radical resection of cervical cancer is the major issue, and there are currently no standard criteria for the success of transversus abdominis plane block anesthesia. Therefore, further analysis to solve these deficiencies is required in future research.

## Data Availability Statement

The raw data supporting the conclusions of this article will be made available by the authors, without undue reservation.

## Ethics Statement

The studies involving human participants were reviewed and approved by Fourth Hospital of Shijiazhuang. The patients/participants provided their written informed consent to participate in this study.

## Author Contributions

All authors listed have made a substantial, direct, and intellectual contribution to the work and approved it for publication.

## Conflict of Interest

The authors declare that the research was conducted in the absence of any commercial or financial relationships that could be construed as a potential conflict of interest.

## Publisher's Note

All claims expressed in this article are solely those of the authors and do not necessarily represent those of their affiliated organizations, or those of the publisher, the editors and the reviewers. Any product that may be evaluated in this article, or claim that may be made by its manufacturer, is not guaranteed or endorsed by the publisher.
